# Biomechanical investigation of an alternative concept to angular stable plating using conventional fixation hardware

**DOI:** 10.1186/1471-2474-11-95

**Published:** 2010-05-21

**Authors:** Markus Windolf, Kajetan Klos, Dirk Wähnert, Bas van der Pol, Roman Radtke, Karsten Schwieger, Roland P Jakob

**Affiliations:** 1 AO Research Institute Davos, AO Foundation, Clavadelerstrasse 8, 7270 Davos, Switzerland; 2 Orthopädie und Traumatologie HFR, Kantonsspital Freiburg, 1708 Freiburg, Switzerland

## Abstract

**Background:**

Angle-stable locking plates have improved the surgical management of fractures. However, locking implants are costly and removal can be difficult. The aim of this in vitro study was to evaluate the biomechanical performance of a newly proposed crossed-screw concept ("Fence") utilizing conventional (non-locked) implants in comparison to conventional LC-DCP (limited contact dynamic compression plate) and LCP (locking compression plate) stabilization, in a human cadaveric diaphyseal gap model.

**Methods:**

In eight pairs of human cadaveric femora, one femur per pair was randomly assigned to receive a Fence construct with either elevated or non-elevated plate, while the contralateral femur received either an LCP or LC-DCP instrumentation. Fracture gap motion and fatigue performance under cyclic loading was evaluated successively in axial compression and in torsion. Results were statistically compared in a pairwise setting.

**Results:**

The elevated Fence constructs allowed significantly higher gap motion compared to the LCP instrumentations (axial compression: p ≤ 0.011, torsion p ≤ 0.015) but revealed similar performance under cyclic loading (p = 0.43). The Fence instrumentation with established bone-plate contact revealed larger fracture gap motion under axial compression compared to the conventional LC-DCP osteosynthesis (p ≤ 0.017). However, all contact Fence specimens survived the cyclic test, whereas all LC-DCP constructs failed early during torsion testing (p < 0.001). All failures occurred due to breakage of the screw heads.

**Conclusions:**

Even though accentuated fracture gap motion became obvious, the "Fence" technique is considered an alternative to cost-intensive locking-head devices. The concept can be of interest in cases were angle-stable implants are unavailable and can lead to new strategies in implant design.

## Background

The devices known as angular stable internal fixators have enhanced the armamentarium for surgical fracture treatment [1-3]. The mechanical principle of these implants is the locking of the screw head into the plate, resulting in a load transfer via plate and screws [1-3]. This increases the stability of the construct and eliminates the risk of loss of reduction due to screw toggling. Furthermore, the periosteal blood supply of the bone under the device is preserved, since there is no need for contact or compression between plate and bone. Biomechanical studies [4-6] have shown the advantages of angle-stable plate fixation over conventional plating. However, several unique complications have been noted, such as difficulty with implant removal and implant cut out in osteoporotic bone [7]. Furthermore, locking implants increase the cost of surgery, which is why many surgeons are restricted in the use of angle-stable fixation hardware. Developing countries and countries with a small budget health care system rarely use these techniques [8,9].

The objective of this study was to compare the biomechanical performance of a newly proposed crossed screw technique ("Fence") utilizing a conventional LC-DCP (limited contact dynamic compression plate) to LCP (locking compression plate) and standard LC-DCP stabilization in a human cadaveric diaphyseal gap model. Fracture gap motion and fatigue properties under cyclic loading were evaluated under axial compression and torsion.

The null hypothesis was that the construct created with conventional screws in a crossed configuration would yield biomechanical results comparable to those achieved with the other instrumentations.

## Methods

### Specimens and study-groups

**Figure 1:**
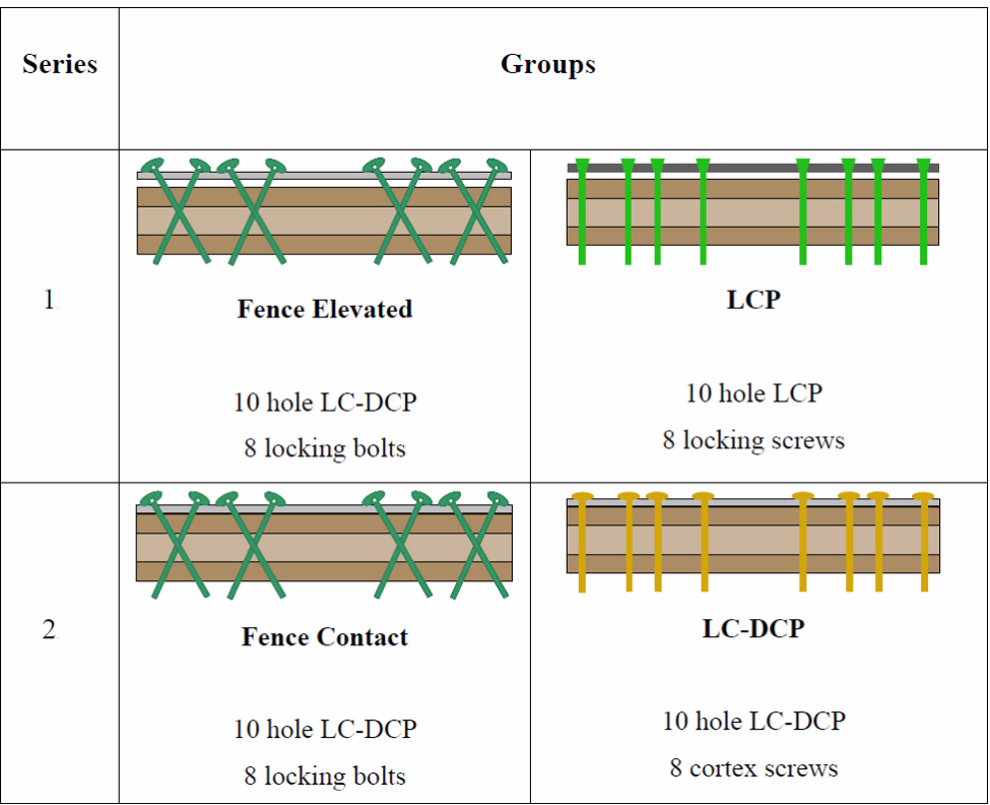
**Study-groups and test-series**. Pairwise comparisons were carried out investigating the elevated Fence construct versus the LCP instrumentation (Series 1) and the contact Fence technique versus the LC-DCP instrumentation (Series 2).

Eight pairs of fresh frozen (-20°C) human cadaveric femora (7 male, 1 female donors; mean donor age 74 years; range 64 - 83 years) were obtained from the department of Pathology, Kantonsspital Basel, Switzerland, where they had been harvested post mortem with appropriate consent of the relatives. Use of the specimens for the purpose of the present study was approved by the ethical commission of Kantonsspital Basel. Soft tissue was removed before instrumentation and mechanical testing. Bone mineral density (BMD) was measured by means of CT-scanning (XtremeCT, SCANCO Medical AG, Bassersdorf, Switzerland) in the cortical bone of the femur diaphysis. The specimens were pairwise assigned to four study-groups according to Figure 1. Within each pair of femora, one femur was randomly assigned to receive a Fence (elevated or contact) construct, while the contralateral femur was assigned to receive an LCP or a conventional LC-DCP instrumentation. Two test-series were established. Series 1: Pairwise comparison between the elevated Fence construct and the LCP instrumentation. Series 2: pairwise comparison between the contact Fence construct and the conventional LC-DCP instrumentation.

### Instrumentations

**Figure 2:**
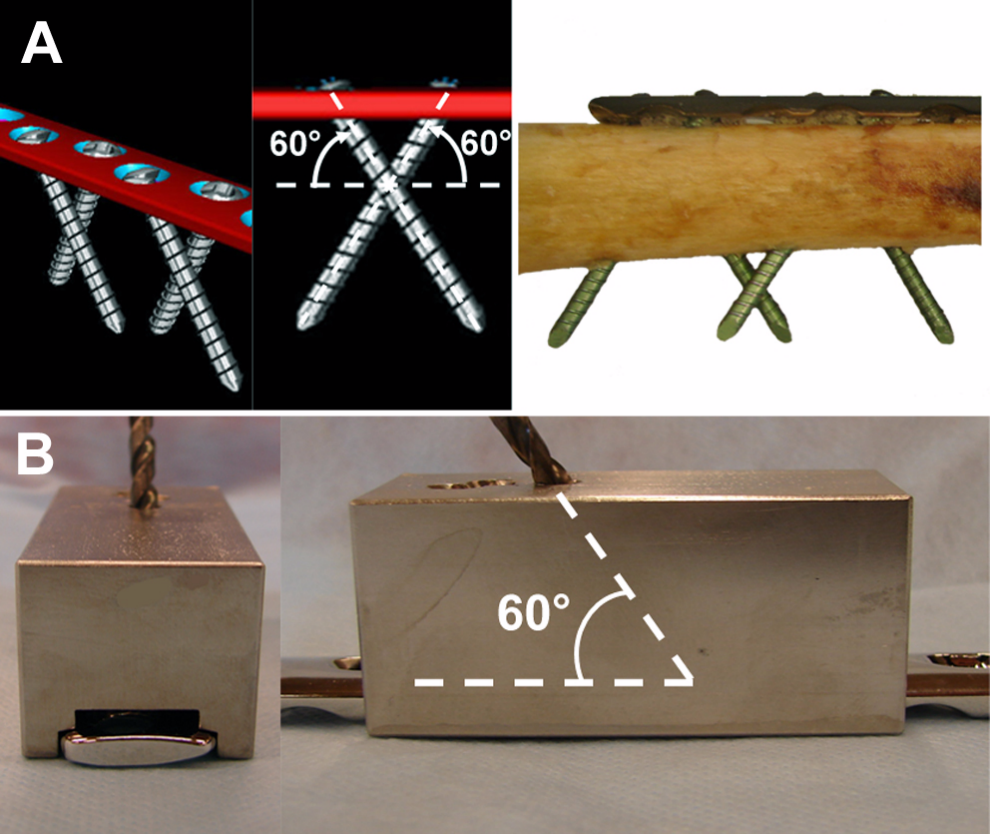
**Fence technique**. Crossed screw pattern with 60° screw angulation (A). A custom-made drill guide was used for standardized instrumentation (B).

**Figure 3:**
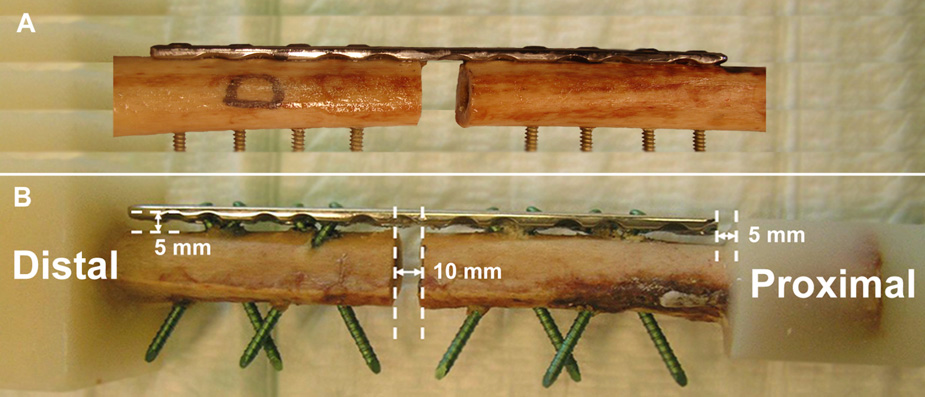
**Fracture model**. A 10-mm mid-diaphyseal gap was created to simulate a severely comminuted fracture. (A) LC-DCP instrumentation. (B) Fence construct with 5 mm elevated plate.

For the Fence constructs, LC-DCP plates (10-hole, 4.5-mm broad LC-DCP) and conventional 4.9-mm self-tapping non-locking head locking bolts were used. Locking bolts were preferred over cortex screws, because of an increased core diameter and altered loading environment. In contrast to conventional compression osteosynthesis, where screws are loaded in tension, shear and bending is expected here. The bolts were routed in a criss-cross pattern resembling a fence, inserted with 60° angulation to the longitudinal shaft axis in pairwise converging bicortical arrangement (Figure 2A). The Fence technique is based on the geometrical principle that the plate can not be displaced along non-parallel screw axes and is therefore constrained. To avoid contact between neighboured screws at the crossing point, a custom-made drill guide was used (Figure 2B). For the elevated Fence constructs (Series 1), the plates were offset from the bone surface by 5-mm-thick plastic spacers (Figure 3B); for the contact Fence constructs (Series 2), the plates were placed directly on the cortex.

For the LCP (Series 1) and the conventional LC-DCP constructs (Series 2), standard plating techniques were used (Figure 3A). The LCP plates (10-hole, 4.5/5.0-mm broad LCP) were attached with 4.9-mm self-tapping head locking screws inserted through the threaded portion of the combination hole provided in the plate. Head locking screws were tightened using a torque limiter. 5-mm-thick spacers were used to offset the plates from the cortex (Figure 3B). The LC-DCP plates were placed directly on the cortex and attached using 4.5-mm self-tapping bicortical cortex screws, since 4.9-mm screws of this type are not available. All conventional screws and bolts were tightened by hand following the clinical practise.

**Figure 4:**
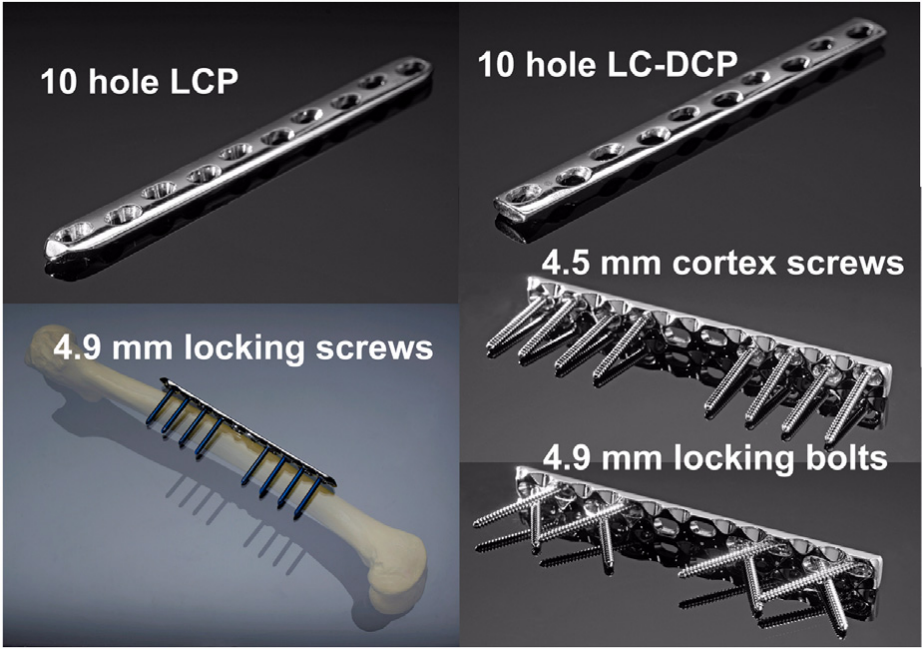
**Implants**. Implants used in the study. Left: 10-hole LCP plate and locking screws. Right: 10-hole LC-DCP plate with conventional and "Fence" screw configuration.

All implants were obtained from the same manufacturer (Synthes GmbH, Bettlach, Switzerland). Plate material was stainless steel; all screws and bolts were made of Titanium. All plates were positioned in the centre of the femoral shaft. A 10-mm transverse osteotomy was created with an oscillating saw below plate holes 5 and 6 to simulate an unstable diaphyseal fracture (Figure 3B). All instrumentations were performed by the same experienced surgeon. The investigated constructs and test-series are visualized in Figure 1. For details of the hardware see Figure 4.

### Mechanical testing

The bones were cut proximally and distally at a distance of 60 mm from the ends of each plate, and potted in Polymethylmethacrylate (PMMA, Beracryl, W. Troller Kunststoffe AG, Jegenstorf, Switzerland). At either end of the plate, a 5-mm distance was ensured between the plate and the potting material (Figure 3B).

**Figure 5:**
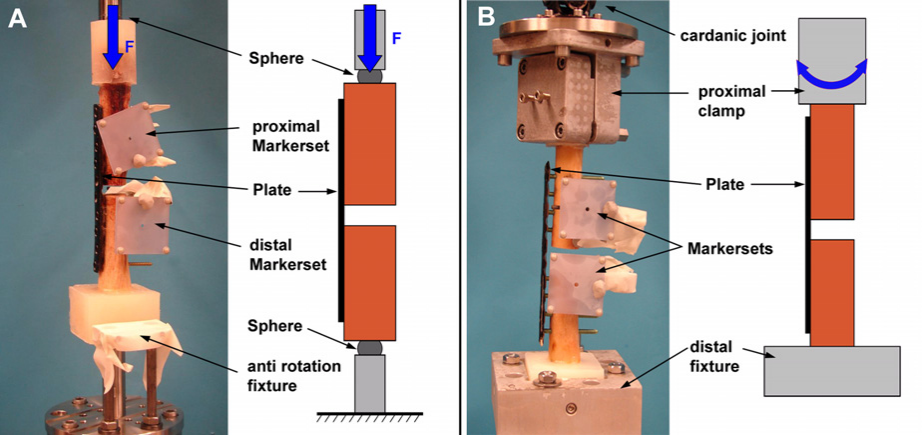
**Test setups**. Setup for the axial compression test including reflective marker-sets for data acquisition. (B) Setup for subsequent torsional testing of the specimens.

Fatigue performances, construct stiffness and fracture gap motion were investigated in a biomechanical experiment consisting of a cyclic axial compression test followed by cyclic torsion until failure of the construct. The test was carried out on a servo-hydraulic test system (858 Mini Bionix^® ^II, MTS Systems Corporation, Eden Prairie, USA) in axial-torsional configuration, equipped with a 25-kN/250-Nm load cell. For the axial compression test the load was proximally introduced via a metal sphere centred on the axis of the femur. A second sphere was located at the distal end of the specimen. The spheres were chosen to replicate the function of the hip and knee joints. The distance between the plate and the centre of the sphere in mediolateral direction was kept constant within each bone pair, so as to ensure a constant lever arm. Sinusoidal axial compression was performed between 100 and 1000 N at 1 Hz for 5000 cycles (Figure 5A). In case no fatal failure occurred, the axial test was continued in cyclic torsion. The proximal sphere was replaced by a double-cardanic joint for the transfer of torque. The distal part was rigidly affixed to the baseplate. Sinusoidal loading was carried out between +20 and -20 Nm at 1 Hz for another 5000 cycles or until construct failure (Figure 5B). The axial load was kept constant at 0 N throughout the torsion test.

### Data acquisition and analysis

**Figure 6:**
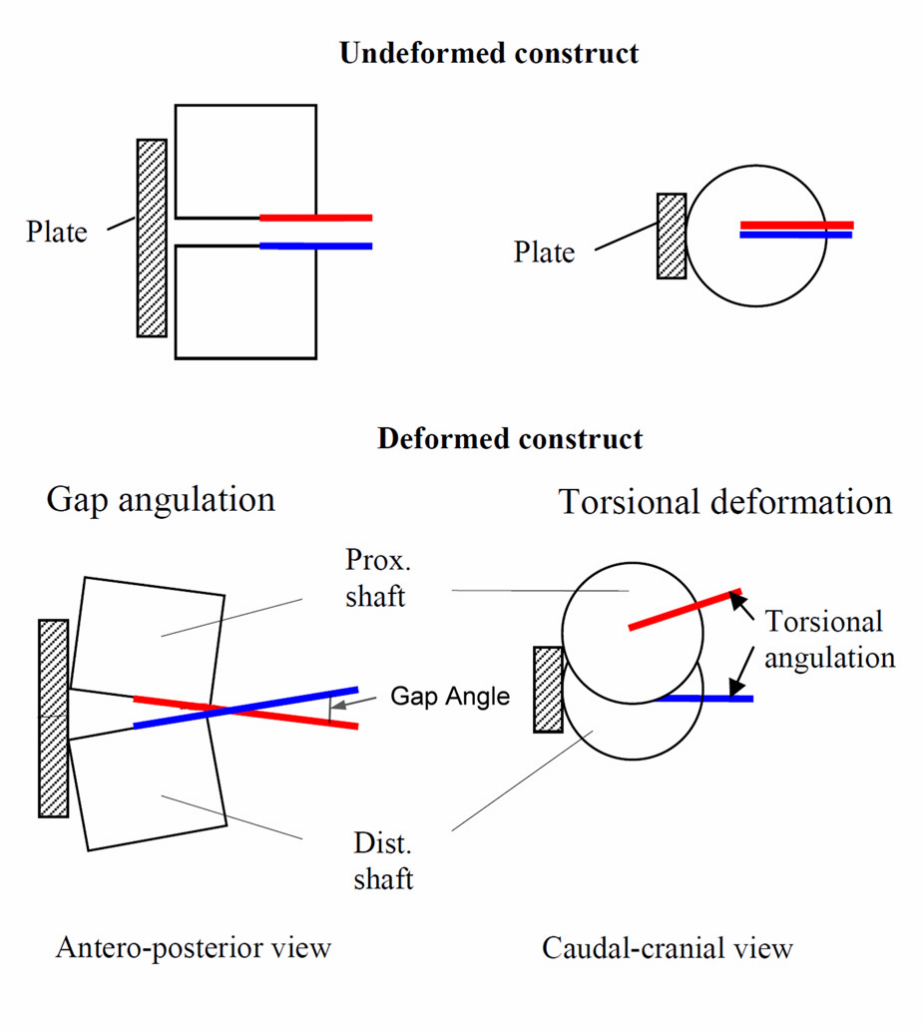
**Data evaluation**. Schematic sketch of axial and torsional deformations as determined from optical motion tracking. Left: definition of the fracture gap angulation as measure for axial deformation. Right: torsional deformation angle.

Displacement, load, angle, and torque were recorded, at a rate of 50 Hz, from the transducers of the test system. Additionally, an optical 3D motion tracking system with five ProReflex MCU digital cameras (Qualisys Motion Capture System; Qualisys AB, Gothenburg, Sweden) was used to identify relative motion in the fracture gap. Reflective marker-sets were attached to the proximal and distal femur shaft fragments (Figure 5). The fracture gap angle under axial loading and the torsional deformation were calculated for all specimens throughout the test (Figure 6). Initial construct stiffness in axial direction was determined at the beginning of the test as change in fracture-gap angulation per unit load. Torsional stiffness was defined as torsional deformation per unit torque at the beginning of the torsion test. Additionally, the range of motion in the fracture gap was defined as amplitude of the gap angle/torsional angle within one load cycle and was evaluated at 1, 2000 and 4000 test cycles for the axial as well as for the torsion test, if applicable. Fatigue performance of the constructs was quantified by the number of load cycles until an angular deformation larger then 15° was reached or when an obvious failure of the osteosynthesis occurred. A threshold of 15° was chosen from pilot experiments using artificial bones.

For comparisons within test-series 1 and 2 (elevated Fence versus LCP; contact Fence versus LC-DCP), paired t-tests were employed on cycles to failure and range of gap motion at 1, 2000 and 4000 cycles. Furthermore, a Repeated Measures ANOVA (analysis of variance) was used to compare between gap motion at 1, 2000 and 4000 cycles within each group. A statistical software package (SPSS 18.0, SPSS Inc., Chicago, USA) was used. Level of significance was set to α = 0.05.

## Results

Cortical bone density was 625 ± 204 mgHA/cm^3 ^(mean ± SD) for the elevated Fence specimens, 612 ± 202 mgHA/cm^3 ^for the LCP samples, 608 ± 70 mgHA/cm^3 ^for the contact Fence group and 585 ± 129 mgHA/cm^3 ^for the LC-DCP specimens. The donor's mean age was 76 years (range 70 - 83 years, 7 male and 1 female).

**Figure 7:**
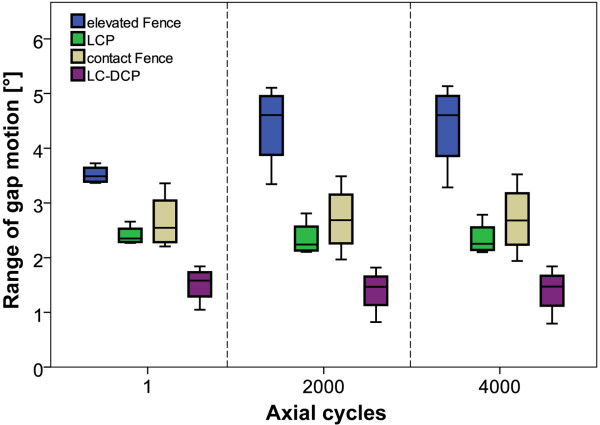
**Axial range of gap motion**. Boxplots of the derived motion in the fracture gap during the cyclic axial test for all study groups. The evaluation was carried out from the motion tracking data at 1, 2000 and 4000 test cycles.

At the beginning of the axial test (cycle 1), the highest gap motion was observed for the elevated Fence specimens (3.52 ± 0.16°, mean ± SD) compared to 2.41 ± 0.18° for the LCP constructs. This difference in gap motion remained statistically significant for all time points (all p ≤ 0.011, Figure 7). The gap motion of the contact Fence group was 2.66 ± 0.51° compared to 1.51 ± 0.33° for the LC-DCP constructs which was the highest observed rigidity in the course of testing. This difference in gap motion was also found statistically significant for all time points (all p ≤ 0.017, Figure 7). When comparing the motion in the fracture gap between time points (1, 2000, 4000 cycles) within each group, no statistical differences were observed (all p ≥ 0.097).

**Figure 8:**
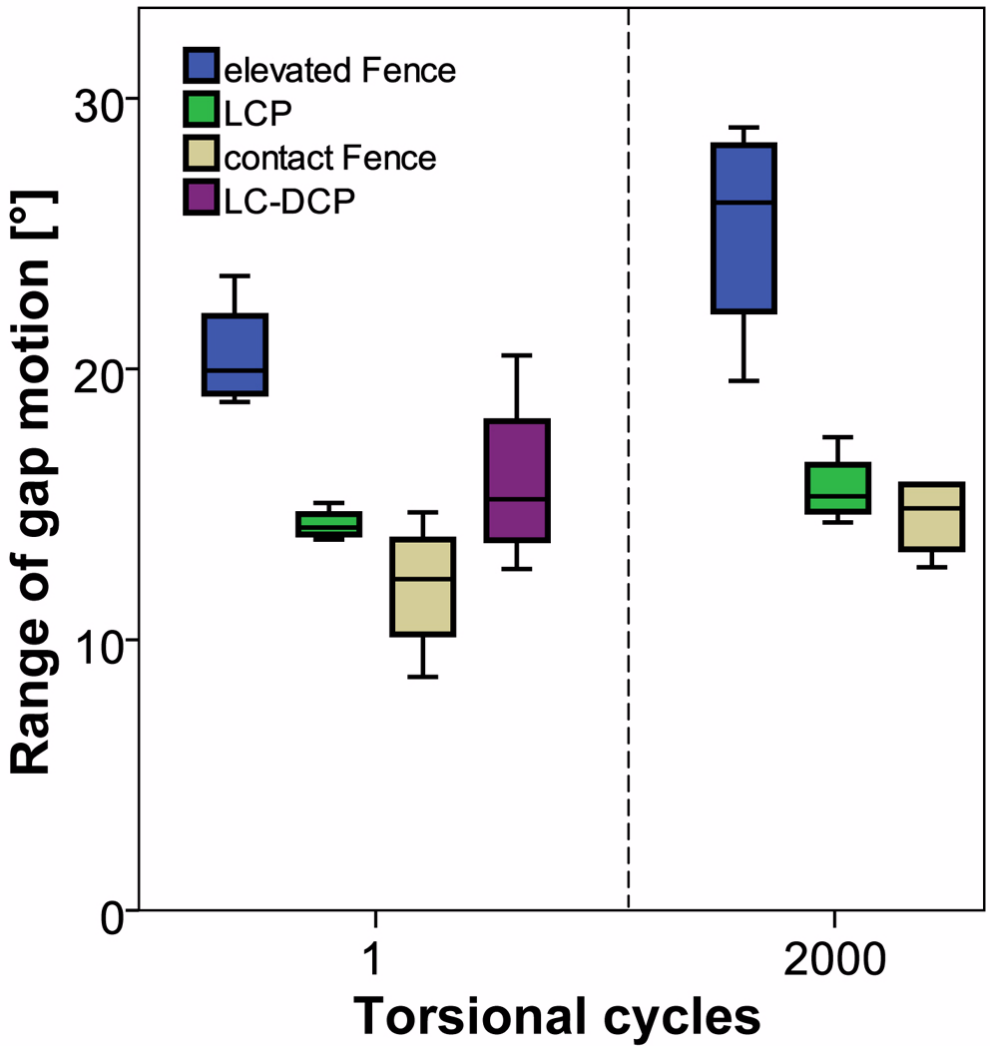
**Torsional range of gap motion**. Boxplots of the derived motion in the fracture gap during the cyclic torsion test for all study groups. The evaluation was carried out from the motion tracking data at 1 and 2000 cycles if applicable. The LC-DCP specimens already failed before the second evaluation step. Comparisons at a later time-point are not feasible.

Regarding torsional gap motion at cycle 1, the elevated Fence constructs revealed again the highest values of the experiment (20.5 ± 2.1°). In comparison, the LCP specimens showed a significantly lower gap motion in torsion of 14.3 ± 0.6° (all p ≤ 0.015, Figure 8). The lowest torsional gap motion at cycle 1 was observed for the contact Fence group (11.9 ± 2.5°) compared to 15.9 ± 3.3° for the LC-DCP specimens. This difference was, however, not significant (p = 0.273, Figure 8). Corresponding values for construct stiffness are shown in Table 1.

**Table 1:** Construct stiffness

	Axial stiffness [N/°]	Torsional stiffness [Nm/°]
elevated Fence	92 ± 25	2.1 ± 0.6
LCP	171 ± 19	2.9 ± 0.1
contact Fence	148 ± 29	3.5 ± 0.4
LC-DCP	299 ± 118	3.0 ± 1.0

Axial and torsional stiffness for all study groups as obtained form a static loading ramp at the beginning of the cyclic axial and torsion tests. Values represent mean ± SD.

**Figure 9:**
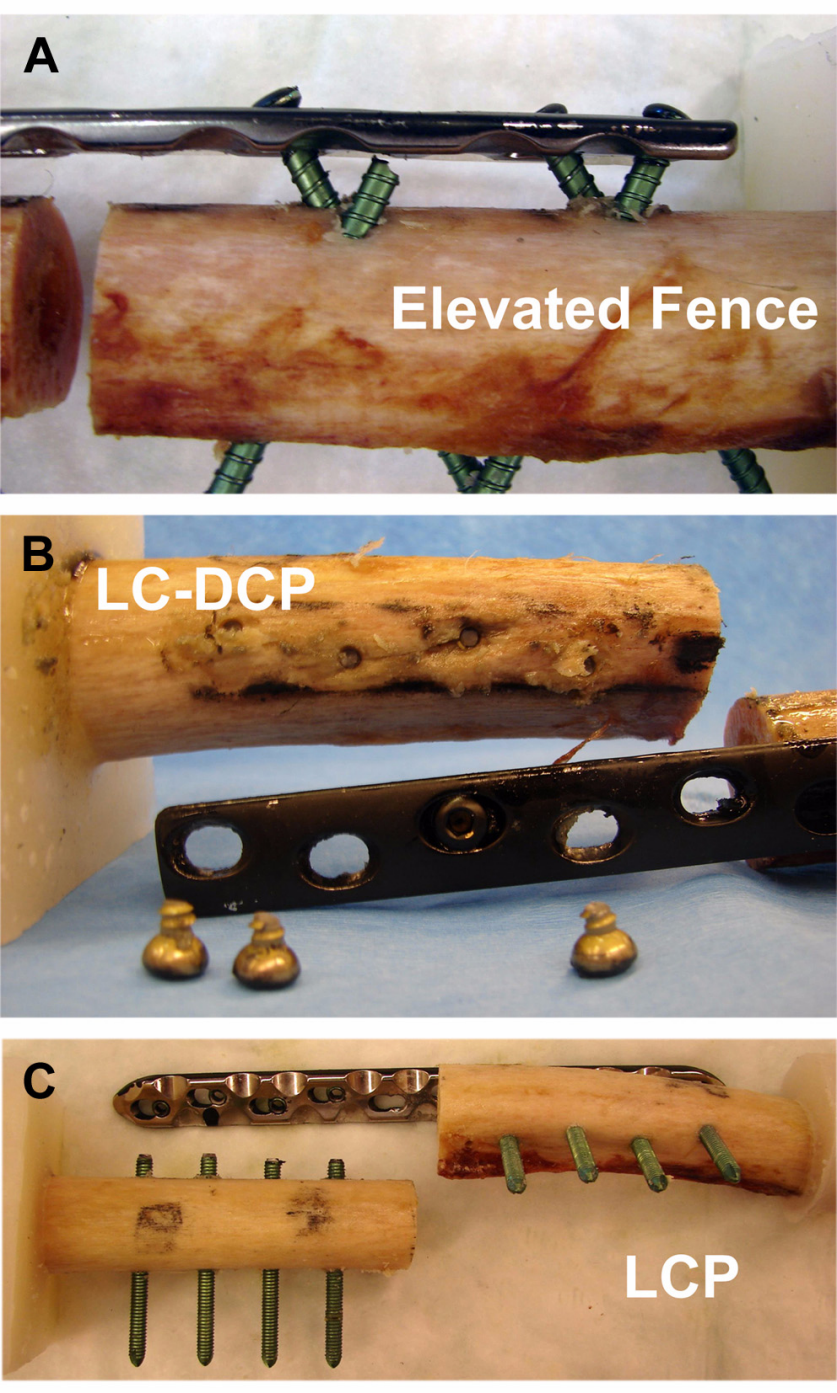
**Failure modes**. Failure modes under cyclic torsional testing for the tested constructs: (A) Elevated Fence, (B) LC-DCP, (C) LCP. No failures occurred in the contact Fence group.

**Figure 10:**
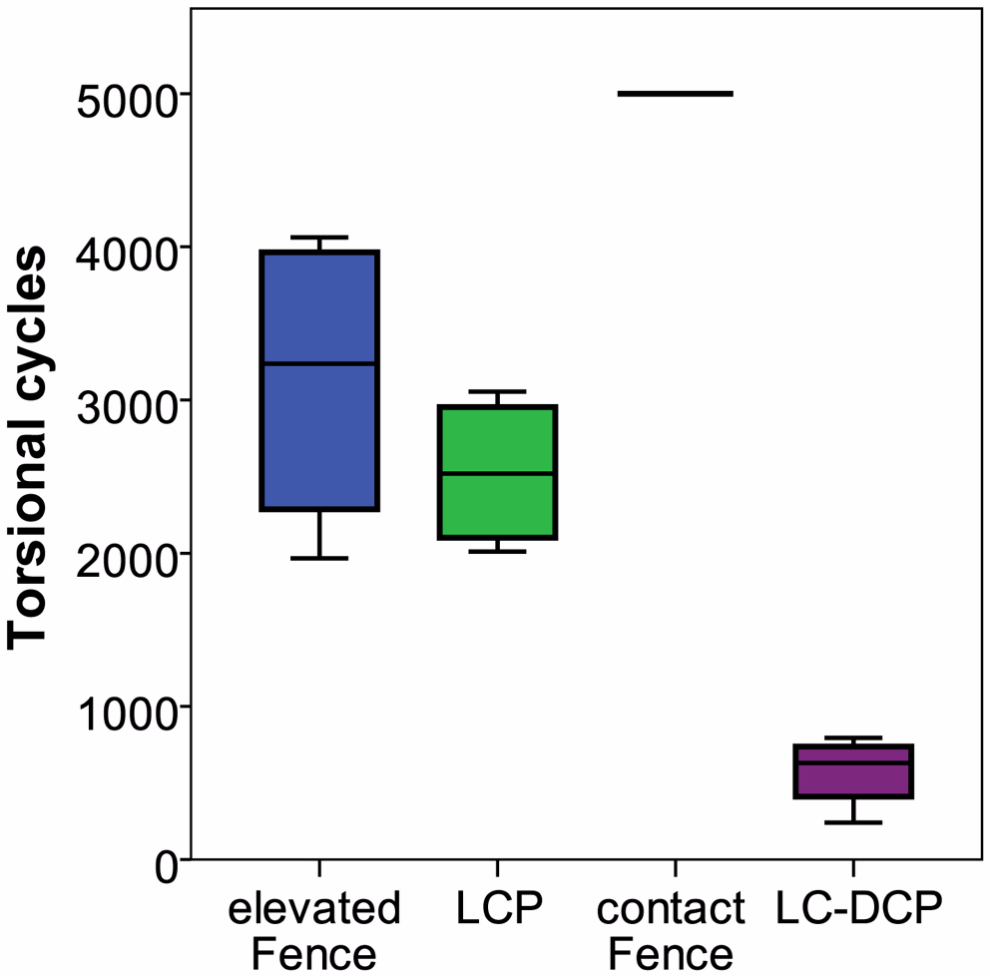
**Cycles to failure**. Boxplots of the number of test cycles until construct failure during cyclic torsional testing. All specimens of the study survived the earlier cyclic compression test. In the contact Fence group no failures occurred in torsion either.

All specimens survived the cyclic test in axial compression. During cyclic torsion, failures occurred due to breakage of the screws at the screw heads (Figure 9 A-C). The elevated Fence and LCP constructs showed similar numbers of load cycles to failure (p = 0.43): 3'125 ± 1'008 for the elevated Fence group and 2'526 ± 505 for the LCP specimens. The LC-DCP constructs failed earliest (574 ± 239). In contrast, all contact Fence instrumentations survived the cyclic torsion test. This difference was statistically significant (p < 0.001, Figure 10).

## Discussion

We investigated the concept of a lower-cost plating technique that would confer the same benefits as those offered by locking plates. The hallmark feature of this technique was the criss-cross pattern of screw routing ("Fence"), using conventional (non-locking-head) locking bolts. An additional advantage of the Fence technique is the variable direction of angulation and screw insertion. Thus, it might be possible to fix additional fragments especially when treating multi-fragmentary fractures. Furthermore, periprosthetic fractures may be addressed using the Fence technique passing the stem of the prosthesis anterior or posterior and at the same time achieving angular stability. The technique with its advantages and opportunities might, however, be more demanding to apply compared to e.g. an LCP instrumentation. A certain experience and skill level of the surgeon is required to avoid complications like screw collisions during implant placement. For further commercialization of the technique an easy-to-use drill template might be an option for ease of the procedure.

In a first step, we compared the Fence technique with established bone contact to conventional, non-locked plating. The conventional constructs were most rigid under axial loading, but failed earliest during cyclic torsional testing, while none of the contact Fence specimens failed. This suggests that the contact Fence technique carries potential to enhance the construct's fatigue properties under cyclic loading conditions compared to conventional plating. However, it has to be taken into account that different screw types with slightly different core diameters were used (cortex screw vs. locking bolt). An influence of this factor can not be excluded. Several authors have investigated the biomechanical properties of locking plates versus conventional plates; findings have been mixed [5,10-14]. Even though not tested in a direct comparison, we found that the LC-DCP constructs failed markedly earlier than did the LCP instrumentations, which would agree with the findings by Lill et al. [15] that flexible constructs are better able to withstand cyclic loading.

In a second test-series, we evaluated a non-contact Fence instrumentation and compared it to an LCP fixation. Both osteosyntheses reflected comparable fatigue properties. However, the Fence technique showed significantly higher fracture gap motion under axial and torsional loading. There is insecurity about the optimal amount of micro-motion in the fracture gap for enhanced bone healing. Hypothetically, a less rigid construct could be advantageous by potentially stimulating callus formation. On the other hand, extensive motion could lead to delayed unions or could cause pseudarthrosis. Although the senior author treated 12 patients successfully with this technique in his trauma centre, further studies-and, in particular, clinical trials-will be required for a definitive assessment of the utility of the technique described in this paper. However, such work would appear to be justified in light of the results of the present study.

Other aspects requiring further investigation might be the screw angulation and the distance of an elevated construct from the cortex of the bone. Ahmad et al. [10] compared LCPs applied at different distances (flush to bone; 2 mm, and 5 mm off the bone), with a DCP control, and found comparable biomechanical behaviour and similar results in the DCP and the LCP constructs in which the plate was applied at or less than 2 mm from the bone. LCP constructs 5 mm off the cortex showed increased plastic deformation and lower failure loads. Similarly, Fulkerson et al. [5], investigating locked-screw constructs, found that increasing the bone-plate distance significantly decreased construct stability. We believe that in our study 5-mm elevation of the plates produced a lever-arm effect at the unsupported free part of the screws which considerably affected the mechanical behaviour of the elevated constructs. Regarding angulation of the Fence pattern, a standardized screw angle of 60° was chosen. The potential effect of this angle on the construct stability was not subject to our investigation. With increasing screw angle the entry points of adjacent screws would approach each other at the near cortex, which could induce a potential weak point. We concluded that fatigue performance and rigidity of the Fence construct may be further optimized by adjusting the bone-plate distance and the screw pattern angulation. Another drawback of the method might be the interdependency within screw pairs. Given only one screw pair is used, failure of one screw would lead to simultaneous loss of stability of the second screw and hence, to failure of the construct.

Our experiment was subject to the limitations common to biomechanical studies. The in vivo loading environment could only be mimicked in a restricted way. We decided to test successively in axial compression and torsion considered as most relevant loading patterns. The sample size was small due to limited availability of bone specimens. We, therefore, decided to carry out only pairwise comparisons without considering the relations between unmatched study-groups. However, conclusions drawn from our findings, based on a low sample size still need to be viewed critically.

## Conclusions

This study introduces a plating technique with crossed screw configuration ("Fence") as a potential alternative to cost-intensive locking-head devices. The fatigue performance was found comparable to angular stable plating, whereas the "Fence" construct allowed larger motion in the fracture gap. A potential influence on bone healing can not be evaluated here. The technique can be of interest in cases were angle-stable implants are unavailable or may lead to new strategies for implant development.

## Competing interests

The authors declare that they have no competing interests.

## Authors' contributions

MW planned the study, carried out the statistics and drafted the manuscript. KK and DW evaluated the data and drafted the manuscript. BV and RR developed the setup and carried out the experiments, KS supported in study planning and helped in the paper draft. RPJ developed the idea of the study and performed the instrumentations. All authors read and approved the final manuscript.

## Pre-publication history

The pre-publication history for this paper can be accessed here:

http://www.biomedcentral.com/1471-2474/11/95/prepub
